# Analysis of Photocatalytic Degradation of Phenol by Zinc Oxide Using Response Surface Methodology

**DOI:** 10.1002/open.202300238

**Published:** 2024-01-09

**Authors:** Meliha Seloglu, Ramazan Orhan, Veyis Selen, Gülbeyi Dursun

**Affiliations:** ^1^ University of Firat Department of Chemical Engineering Elazıg Türkiye; ^2^ University of Firat Department of Bioengineering Elazıg Türkiye

**Keywords:** ZnO, Photocatalytic degradation, Response surface methodology, Phenol, Degradation mechanism

## Abstract

In this study, the photocatalytic degradation of phenol, which is commonly found in industrial wastewater at high rates, was investigated using a zinc oxide (ZnO) catalyst. It is thought that our findings will contribute to the removal of phenol in industrial wastewater. The experimental study was conducted in a batch‐type air‐fed cylindrical photocatalytic reactor, and a central composite design (CCD) was chosen and analyzed using response surface methodology (RSM). The study aimed to explore the effects of initial phenol concentration, catalyst concentration, airflow rate, and degradation time on the photocatalytic degradation of phenol and the removal efficiency of total organic carbon (TOC). A quadratic regression model was developed to establish the relationship between phenol degradation, TOC removal effectiveness, and the four factors mentioned. The validity of the model was assessed through an analysis of variance (ANOVA). A good agreement was observed between the model results and the experimental data. As a result of the experiments carried out under optimized conditions, the degradation percentage of phenol was found to be 77.15 %, and the degradation percentage of TOC was 59.87 %. Additionally, pseudo‐first‐order kinetics were used in the photocatalytic degradation of phenol.

## Graphical abstract

Phenol and phenolic compounds pose a serious threat to the ecosystem, human health, and water resources. Photocatalytic degradation is the most suitable technique for removing organic pollutants from wastewater, and ZnO is an effective photocatalyst. This study evaluated both the photocatalytic degradation of phenol and the measurement of TOC using a ZnO photocatalyst, demonstrating the feasibility of employing RSM.

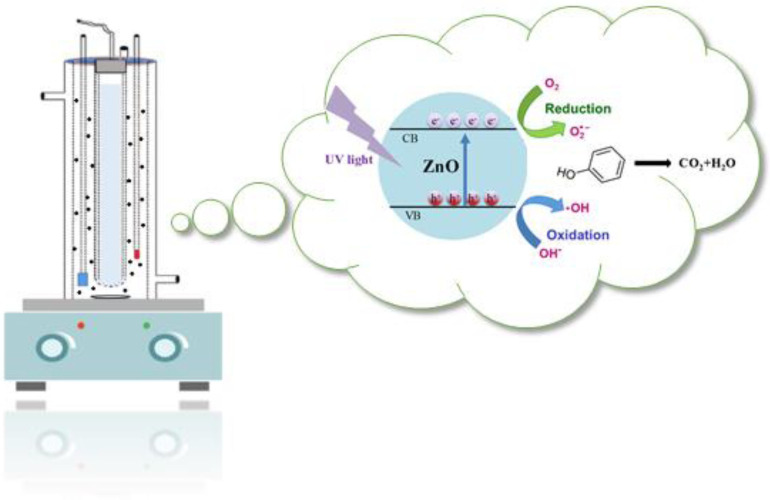

## Introduction

Water is accepted as the most valuable natural resource necessary for the survival of all living things. Ensuring easy access to clean water stands as one of humanity‘s paramount concerns. As a result of increasing industrialization and urbanization in the world in the last few decades, and again according to a recent report of the United Nations, it is predicted that both population and chemical production will increase.^[1]^ It has been reported by Akhtar et al. that this industrial growth plays an important role in the depletion and pollution of freshwater resources and that this problem will continue to increase in the coming years.^[2]^

Advances in science, technology, and industrialization have provided significant benefits to humanity, but the continuous discharge of toxic organic pollutants into the aquatic environment without any appropriate treatment causes pollution of water and the environmental at worrying levels.^[3]^ Dyes, surfactants, heavy metals, pharmaceuticals, and other organic pollutants are various contaminants in water resources. Among these water‐soluble organic pollutants, the global yearly production of phenol and its derivatives is approximately 3 million tons.^[4]^

Phenol and phenolic compounds (chlorophenols, nitrophenols, etc.) present a significant threat to ecosystems, human health, and water resources due to their toxicity, endocrine‐disrupting capabilities, and carcinogenic effects.^[4]^ The United States, the European Union, and Canada have listed phenol and a few related chemicals as priority pollutants.[^5^, ^6^] Phenol is widely used in the production of phenolic resins, caprolactam, bisphenol A, and chlorophenols, such as pentachlorophenol.^[4]^ Phenol and its derivatives have been determined in effluents from petroleum refining,^[7]^ paper and pulp manufacturing,^[8]^ coal processing^[9]^ and chemical production facilities.^[10]^ Therefore, there is a growing need to develop effective treatment technologies for the abatement of organic pollutants from wastewater, including phenols and other pollutants, for human consumption and aquatic life.^[11]^

Physical, chemical, and biological treatment techniques are used to remove phenol from wastewater. Membrane filtration, flotation, coagulation‐flocculation, adsorption, precipitation, and ion exchange[^12^, ^13^, ^14^, ^15^, ^16^, ^17^] are used as physico‐chemical treatment techniques, while aerobic and anaerobic processes, bacterial and fungal biosorption[^18^, ^19^] are used as biological treatment techniques. However, there are many limitations in these processes, such as high cost and low efficiency, and these processes cannot entirely remove phenolic compounds from wastewater.[^20^, ^21^] Additionally, these existing methods have disadvantages such as the generation of new waste during the treatment process and requiring extra costs for further treatment steps.^[22]^ For this reason, it is necessary to develop and prefer inexpensive methods that will be alternatives to the existing methods. As an alternative to the methods mentioned, “photocatalytic degradation” has been developed as a non‐toxic, highly efficient, and low‐cost technique. Akhtar et al. stated in their study that the photolytic and photocatalytic degradation processes are currently considered the most promising techniques used in wide areas of applications.^[2]^ This method involves the conversion of harmful organic pollutants in wastewater into harmless products such as water and carbon dioxide by “decomposing” with an activated semiconductor by using UV light.[^23^, ^24^, ^25^] TiO_2_ is a widely used photocatalyst in the photocatalytic degradation of organic pollutants, but it is not economical for large‐scale water treatment operations. However, it has been reported in various studies that ZnO is more effective than TiO_2_ in the photocatalytic degradation of organic pollutants. ZnO photocatalyst has also been suggested as a suitable alternative to TiO_2_ due to its strong oxidation ability, wide and direct band gap of 3.37 eV at room temperature, excellent piezoelectric effect, low cost, non‐toxicity, and high free‐exciton binding energy of 60 meV at room temperature. It adsorbs a large fraction of UV radiation.[^26^, ^27^, ^28^, ^29^, ^30^]

The morphology and/or chemistry of the catalyst, as well as the operating settings, can all be optimized to enhance removal efficiency. The efficiency of the photocatalytic degradation process depends on the concentration of pollutant and photocatalyst, time, airflow rate, etc. parameters. As a result, optimizing these parameters is crucial to achieving photocatalytic degradation of the existing pollutant. One of the most extensively used optimization techniques for this purpose is response surface methodology (RSM).^[31]^ RSM is a set of statistical and mathematical techniques used to improve current process design, as well as experimental design and process optimization. By systematically altering all the variables at once, it has been acknowledged as the most trustworthy and empirical statistical method used to assess the impact of different process parameters on photocatalytic degradation.[^32^, ^33^] Furthermore, by developing a mathematical model (linear, square polynomial functions, etc.), it is possible to determine the degree of effect of each parameter on process efficiency and to investigate the interaction between them.^[34]^ The most significant benefit of using RSM is that it provides a systematic method for designing tests that generally require fewer experiments and, as a result, less time. To design these experiments, a Box‐Benkhen Design (BBD) and a Central Composite Design (CCD) are widely used for optimization.[^20^, ^35^] In our study, CCD was preferred as the design approach.

In this study, the degradation of phenol in a photocatalytic reactor using ZnO photocatalyst was investigated. The effects of operating parameters such as photocatalyst concentration, initial phenol concentration, airflow rate, and degradation time on photocatalytic degradation efficiency were determined, and total organic carbon (TOC) removal was investigated together with phenol removal. To examine the phenol and TOC removal effects, process parameters were optimized using RSM, and a second‐order regression model was developed to estimate the removal percentages.

## Results and Discussion

### Photocatalyst Characterization

**Figure 1:**
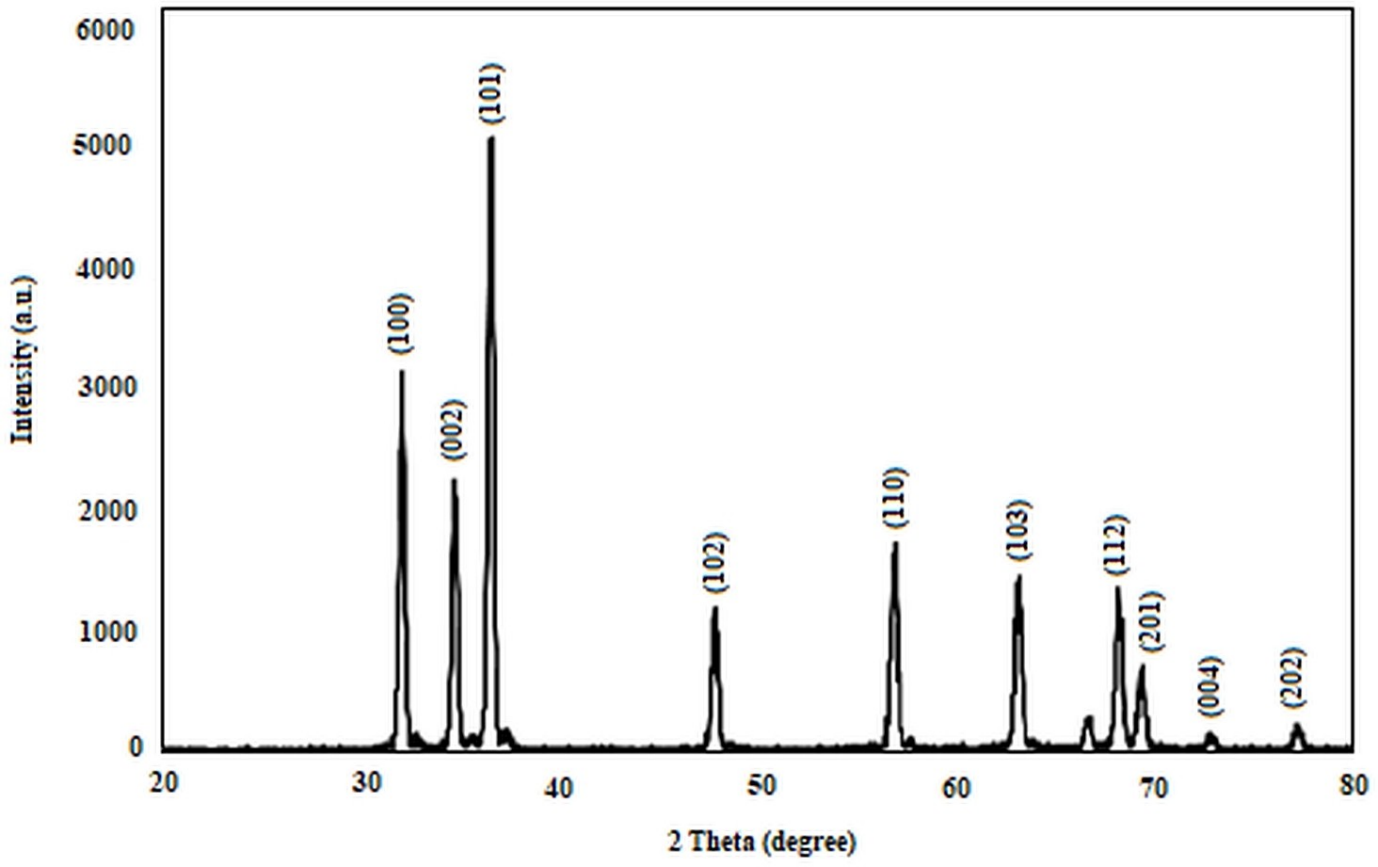
The XRD pattern of zinc oxide hexagonal phase.

The XRD pattern of ZnO is illustrated in Figure 1. The XRD patterns show noticeable peaks corresponding to 2θ values of 31.87°, 34.45°, 36.21°, 47.75°, 56.77°, 63.04°, 68.1°, 69.23°, 72.73°, and 77.13°. The prominent peaks correspond to values of (100), (002), (101), (102), (110), (103), (112), (201), (004) and (202). All XRD diffraction peaks of ZnO powders were consistent with the wurtzite structure as reported in the JCPDS data,^[36]^ and no characteristic peaks were observed other than ZnO.

**Figure 2:**
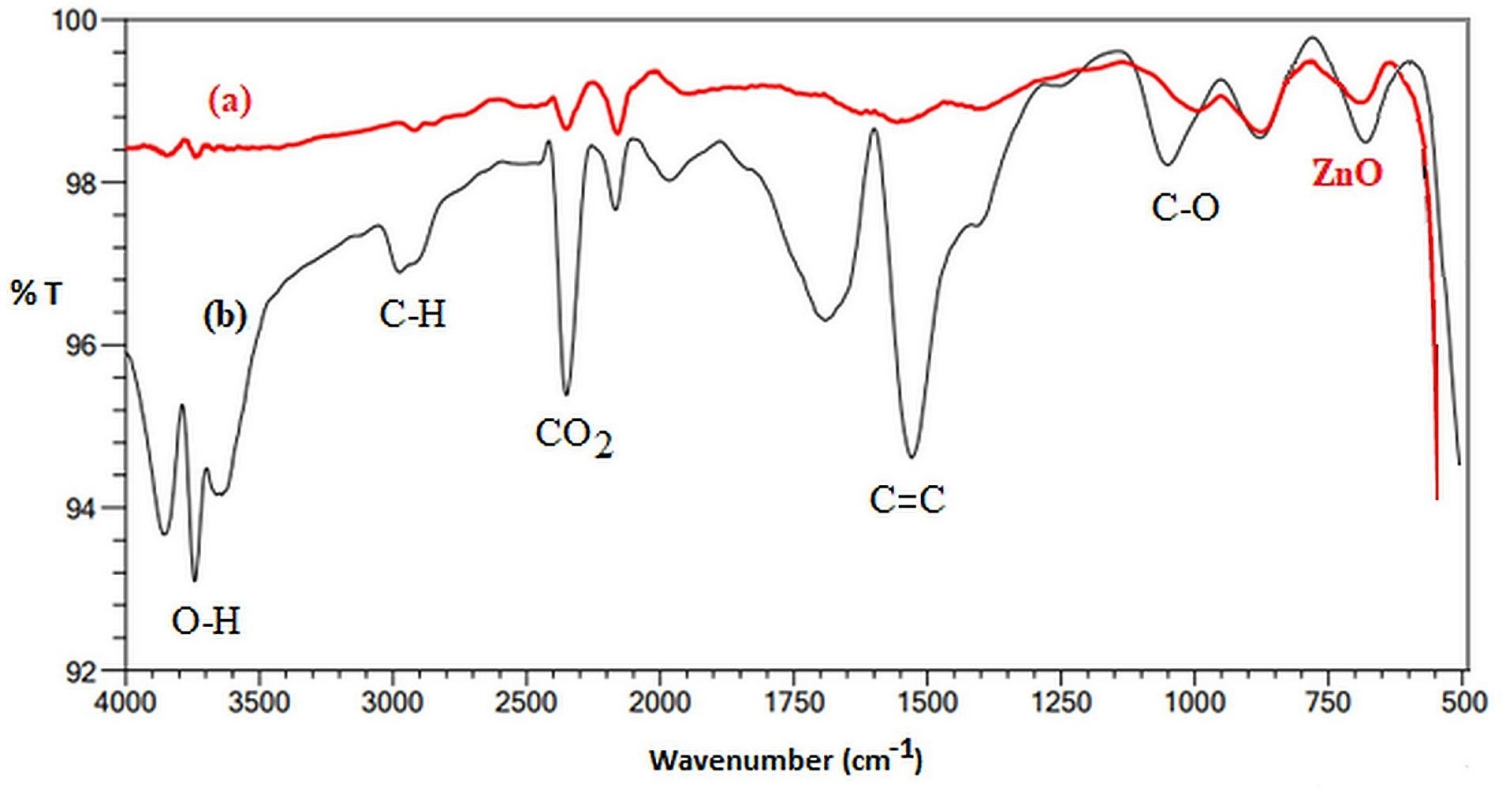
FTIR spectra of ZnO at time t=0 (a) and after t=135 min (b).

FTIR spectra and bond stretching vibrations in the range of 500–4000 cm^−1^ of the ZnO before adsorbed phenol solution at t=0 and after the degradation process in the photocatalytic reactor for t=135 min. are given in Figure 2 (a and b), respectively. The peaks in Figure 2 a can be explained as follows: the spectra in the range 500 and 900 cm^−1^ are associated with the characteristic zinc‐oxygen (Zn−O) stretching vibration mode because metal bands are generally observed below 1000 cm^−1^. The absorption peaks at 3850 and 3400 cm^−1^ are due to the stretching mode of the O−H group, which reveals the existence of water absorbed by the ZnO. The peaks at 2100 and 2500 cm^−1^ appear due to the atmospheric CO_2_ present in the instrument.[^37^, ^38^, ^39^] It can be seen from Figure 2 b that new absorption bands in the region of 1000–3100 cm^−1^ appeared on the spectra of ZnO used as a result of the degradation of phenol in the photocatalytic reactor. These bands are observed due to aromatic ring vibrations in the structure of phenol, i. e., the (C−H) at 2980 cm^−1^, the (C=C) at 1530 cm^−1^, and the (C−O) at 1050 cm^−1^.[^40^, ^41^] When these peaks in Figure 2 are compared, the appearance of some peaks in ZnO after the degradation process (Figure 2b) shows that the phenol in the solution has been removed.

### Model development and statistical analysis

The RSM was conducted in 30 experiments using a four‐variable CCD at each design level of 4 different variants to optimize the degradation of phenol and TOC removal. The conditions of 30 experimental studies, the experimental and estimated values of phenol and TOC as percent removal are given in Table 5. Phenol degradation yield was found in the range of 4.97–97.99 %, and TOC removal (%) was found in the range of 4.99 %–62.66 %. To express the relationship between phenol degradation (%) and process parameters in terms of coded factors (Eq. 1), a quadratic polynomial equation was derived using the data in Table 5.
(1)

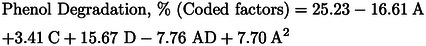




In the following, a second‐order polynomial equation was similarly obtained to express the relationship between TOC removal (%) and process parameters in terms of coded factors (Eq. 2.
(2)

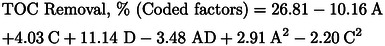




The adequacy and importance of the proposed model were analyzed using the ANOVA test, and the results are shown in Tables 1 and 2. The results of the ANOVA showed that the experimental findings and the anticipated values of the removal efficiencies of phenol and TOC were in good agreement. In terms of statistics, a low p‐value (0.05) and a high F‐value denote the significance of a model or parameter.^[42]^ Therefore, in the interpretation of ANOVA results, it is known that independent variables with a P‐value less than 0.05 are effective on the model expression, whereas independent variables with a P‐value greater than 0.1 are not effective in the model. In the degradation process where the response variable was chosen as TOC removal, the P‐value of catalyst concentration (B), one of the independent variables whose effects were examined, was found to be 0.0813. Since this value was close to 0.05, it was accepted that the catalyst concentration was an effective independent variable in TOC removal. The degree of significance and effectiveness of the recommended response model were determined by the F‐value, P‐value, R^2^, and adjusted R^2^. The Model F‐values of 90.48 and 64.57 obtained for phenol degradation and TOC removal, respectively, in this experiment show that the model is significant. The suitability of the suggested model for the experimental results can be evaluated by considering the correlation coefficient (R^2^) value formed at 0 and 1.

**Table 1:** Analysis of variance of the second‐order polynomial for phenol degradation.

Source	Sum of squares	DF	Mean square	*F*‐value	*P*‐value
Model	15432.83	5	3086.57	90.48	<0.0001
A‐ Initial phenol concentration	6623.4	1	6623.4	194.17	<0.0001
C‐ Airflow rate	279.48	1	279.48	8.19	0.0086
D‐ Degradation time	5896.93	1	5896.93	172.87	<0.0001
AD	964.10	1	964.10	28.26	<0.0001
A^2^	1668.90	1	1668.90	48.93	<0.0001
Residual	818.67	24	34.11		
Lack of fit	807.93	19	42.52	19.79	0.0018
Pure error	10.74	5	2.15		
Cor total	16251.5	29			
R^2^	0.9496				
R^2^_Adj_	0.9391				
R^2^_Pre_	0.8947				
Std. Dev.	5.84				
C.V. %	18.25				
PRESS	1712.07				
Adequate precision	36.38				

**Table 2:** Analysis of variance of the second‐order polynomial for TOC removal.

Source	Sum of squares	DF	Mean square	*F*‐value	*P*‐value
Model	6537.79	9	726.42	64.57	<0.0001
A‐ Initial phenol concentration	2476.8	1	2476.8	220.16	<0.0001
B‐ Catalyst concentration	37.93	1	37.93	3.37	0.0813
C‐ Airflow rate	389.86	1	389.86	34.65	<0.0001
D‐ Degradation time	2981.06	1	2981.06	264.98	<0.0001
AB	0.44	1	0.44	0.039	0.8454
AC	38.22	1	38.22	3.40	0.0802
AD	193.42	1	193.42	17.19	0.0005
A^2^	231.57	1	231.57	19.33	0.0003
C^2^	133.28	1	133.28	13.90	0.0013
Residual	225.0	20	11.25		
Lack of fit	212.88	15	14.19	5.86	0.0305
Pure error	12.12	5	2.42		
Cor total	6762.79	29			
R^2^	0.9667				
R^2^_Adj_	0.9518				
R^2^_Pre_	0.9024				
Std. Dev.	3.35				
C.V. %	11.87				
PRESS	659.80				
Adequate precision	28.138				

**Figure 3:**
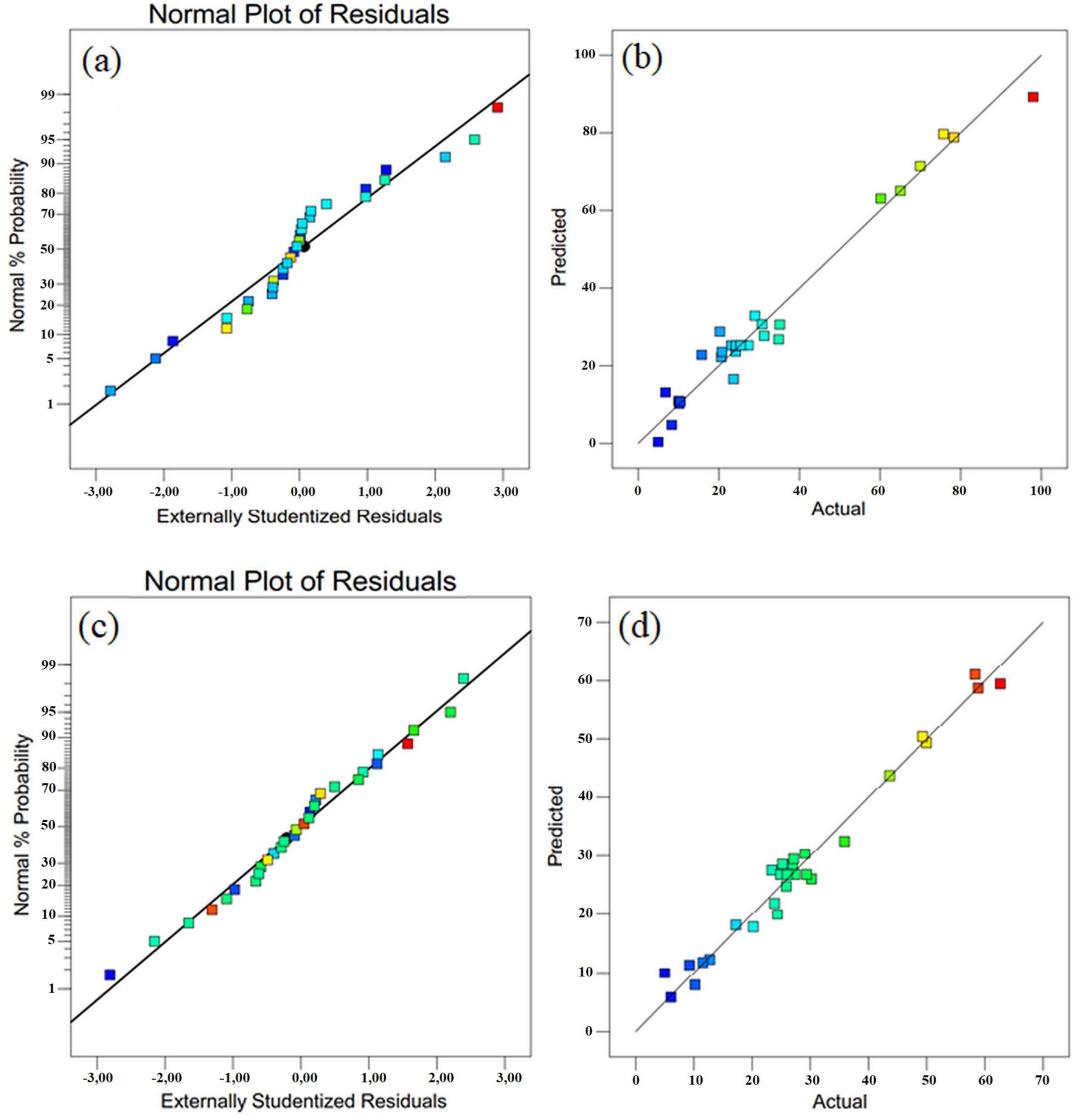
Normal plot of residuals and Predicted vs. actual values for photocatalytic phenol degradation [(a) & (b)] and TOC removal [(c) & (d)].

The regression equation better fits the experimental data when the value of R^2^ approaches one. Since the R^2^ values for phenol degradation and TOC removal are 0.9496 and 0.9667, respectively, it is seen that 95 % of the variations in the dependent variable are defined by the independent variables. High R^2^ values can be obtained by adding variables to the statistically proposed model, but adjusted R^2^ cannot be consequently enhanced, and its use is recommended for the suitability of the model.^[43]^ The adjusted R^2^ values for phenol degradation and TOC removal were found to be 0.9391 and 0.9518, respectively. For the used model, the predicted R^2^ values and the adjusted R^2^ values were close enough to each other. This indicates the adequacy of the proposed model. The low values of the coefficient of variance (C.V), which expresses how much the standard deviation changes as a percentage compared to the mean, shows that the data have the same value as the mean.^[44]^ The coefficients of variance (C.V) obtained for phenol degradation and TOC removal were relatively low values of 18.25 % and 11.87 %, respectively. This shows the accuracy and reliability of the experiments performed. It was possible to determine the signal‐to‐noise ratio with sufficient accuracy, and it is an acceptable value for this ratio to be greater than 4.^[3]^ In our study, these ratios for phenol degradation and TOC removal were found to be 36.38 and 28.14, respectively. This ratio reflects the intended signal‐to‐noise ratio for the quadratic model. P‐values under 0.0500 showed a strong correlation between the process variables and the response. Figure 3 displays the predicted vs. actual response as well as the normal plot of residuals. The fact that the residuals fell in a straight line indicated that the errors were distributed properly.

**Figure 4:**
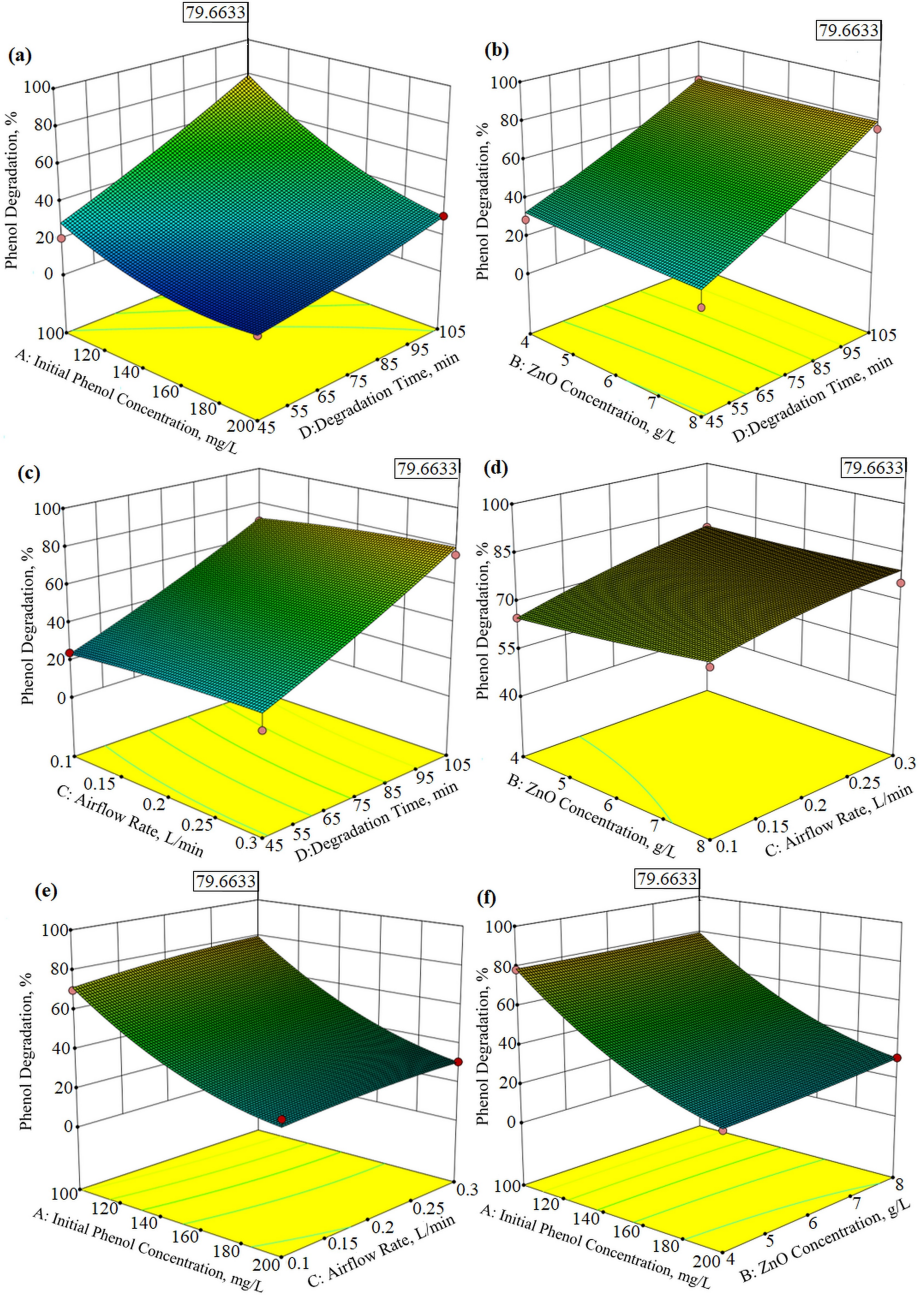
Response Surface Methodology 3D surface plots for phenol degradation as a function of four different parameters.

**Figure 5:**
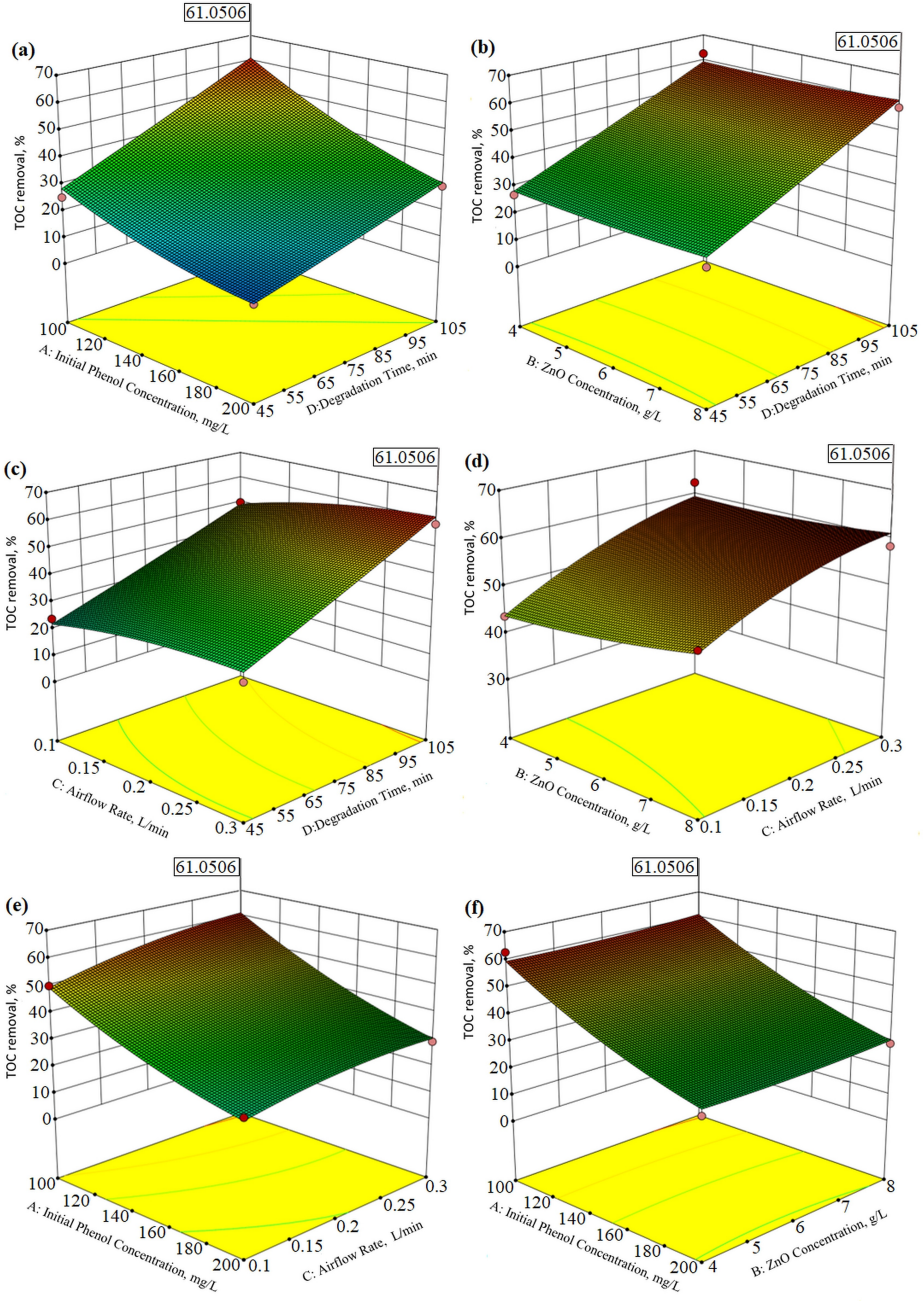
Response Surface Methodology 3D surface plots for TOC removal as a function of four different parameters.

Three‐dimensional surface plots of the effect of four independent parameters such as initial phenol concentration, catalyst concentration, airflow rate, and degradation time for phenol degradation and TOC removal were given in Figures 4 (a–f) and Figures 5 (a–f). These graphs can be interpreted as graphical representations of the regression model equation used to resolve the optimum conditions of the factors, and are usually applied to prove the understanding of the types of interactions between the variables that can be used to improve the removal efficiency. From the curvatures of the 3D plots in Figure 4 (a–f), it is seen that the phenol degradation efficiency decreases with an increase in the initial phenol concentration but increases with an increase in the degradation time and airflow rate. Phenol molecules were loaded onto the photocatalyst‘s surface with a rise in phenol concentration, as phenol molecules in that area retrieved OH* radicals. The formation of OH^*^ radicals and $O_{2}^{-}$ was reduced, which minimizes phenol degradation at high concentrations.^[43]^ Additionally, it is also seen that the catalyst concentration does not have much effect on the phenol degradation efficiency in the given range. The effect of these 4 different parameters on the TOC removal efficiency showed a similar trend as in the phenol degradation efficiency (Figure 5 (a–f)). It was observed that the functional groups were fragmented and the TOC amount was higher at the initial phenol concentration of 100 mg/L. It can be seen from Figure 5 (a–f) that the TOC removal efficiency increases with the increase in degradation time and airflow rate, but does not change with the increase in catalyst concentration.

Numerical optimization includes considering the values of the independent parameters at which the response reaches its maximum, which can be the maximum or minimum. Therefore, a numerical method given by Meyers and Montgomery was used to solve the regression equations (4) and (6).^[45]^ The Design Expert 10 software recommended a series of solutions in the specified range as a result of the carried out experiments in the degradation of phenol and TOC from solution media by a photocatalytic system. Among these solutions, the optimum conditions giving the maximum phenol and TOC degradation yield were chosen. The optimum values giving both the maximum phenol degradation yield (79.66 %) and TOC degradation (61.05 %) proposed by the software were as follows; Initial phenol concentration, 100 mg/L; Catalyst concentration, 8 g/L; Airflow rate, 0.3 L/min; Degradation time, 105 min. The phenol degradation yield and TOC degradation obtained experimentally under optimal conditions were found to be 77.15 % and 59.87 %, respectively. Therefore, there was good agreement between the anticipated and experimental outcomes of the constructed model for both the degradation percentage of phenol (77.15 %) and the degradation percentage of TOC (59.87 %) using the photocatalytic degradation process.

The photocatalytic performance of ZnO used in the study was compared with the literature and is given in Table 3.

**Table 3:** Comparison of the photocatalytic activity of ZnO with literature.

Catalyst	Degradation Efficiency, %	Catalyst Dose, g/L	Initial Concentration, g/L	Reference
ZnO	78.20	1.5	0.025	[46]
Synthesized ZnO	60.00	1.5	0.200	[47]
ZnO‐rGO	84.20	0.2	0.020	[48]
ZnO nanoparticle	95.41	0.2	‐	[49]
ZnO	88.00	0.6	‐	[50]
ZnO	75.00	1.0	0.015	[51]
ZnO	92.30	0.1	0.020	[52]
ZnO	77.15	8.0	0.100	This study

### Kinetics of Photocatalytic Oxidation

In the photocatalytic decomposition reaction, the pollutant adsorbed on the photocatalyst surface or brought with it by mass transfer due to mixing is decomposed by different oxidizing species, such as hydroxyl radical. The photocatalytic degradation rate of various organic pollutants is well described by the Langmuir‐Hinshelwood (L−H) kinetic model derived from the Langmuir adsorption isotherm equation.[^52^, ^53^, ^54^] The results are shown in Table 4. The nonlinear L−H kinetic model is given in Eq. 5.

(5) $$r=\frac{dC}{dt}=\frac{k_{r}K_{s}C}{1+K_{s}C+K_{w}C_{w}}$$

**Table 4:** Results of Langmuir‐Hinshelwood kinetic study.

T (°C)	First‐order kinetic equations	R^2^
10 20 30 40	y=0.0120x−0.0347 y=0.0114x−0.1057 y=0.0104x−0.1102 y=0.0087x−0.0934	0.9924 0.9933 0.9954 0.9886

Here; r; phenol degradation rate (mmol/L.h), C_o_; phenol initial concentration (mmol/L), C: phenol concentration at any time t (mmol/L), t; time (min), k: reaction rate constant (1/min), K_s_: adsorption coefficient of phenol on photocatalyst (ZnO) particle (L/mg), K_w_: water adsorption constant, C_w_: water amount.

Since the amount of water remained practically unchanged, Eq. (6) was obtained by subtracting the term K_w_C_w_ in the denominator from Eq. 5.

(6) $$r=\frac{dC}{dt}=\frac{k_{r}K_{s}C}{1+K_{s}C}$$

Since the adsorption of phenol on the surface of ZnO particles is limited and low in the experiments carried out, a first‐order equation as in Eq. (7) can be obtained by subtracting K_s_C from Eq. 8.

(7) $$r=\frac{dC}{dt}=k_{r}K_{s}C=k_{app}C\;$$

After the integration of Eq. (7), Eq. (8) is obtained.

(8) $$ln\frac{C_{o}}{C}=k_{r}K_{s}t=k_{app}t$$

k_app_ is obtained from the slope of the line obtained from the ln (C_o_/C) versus the t plot created with the data obtained from the experiments against time.

**Figure 6:**
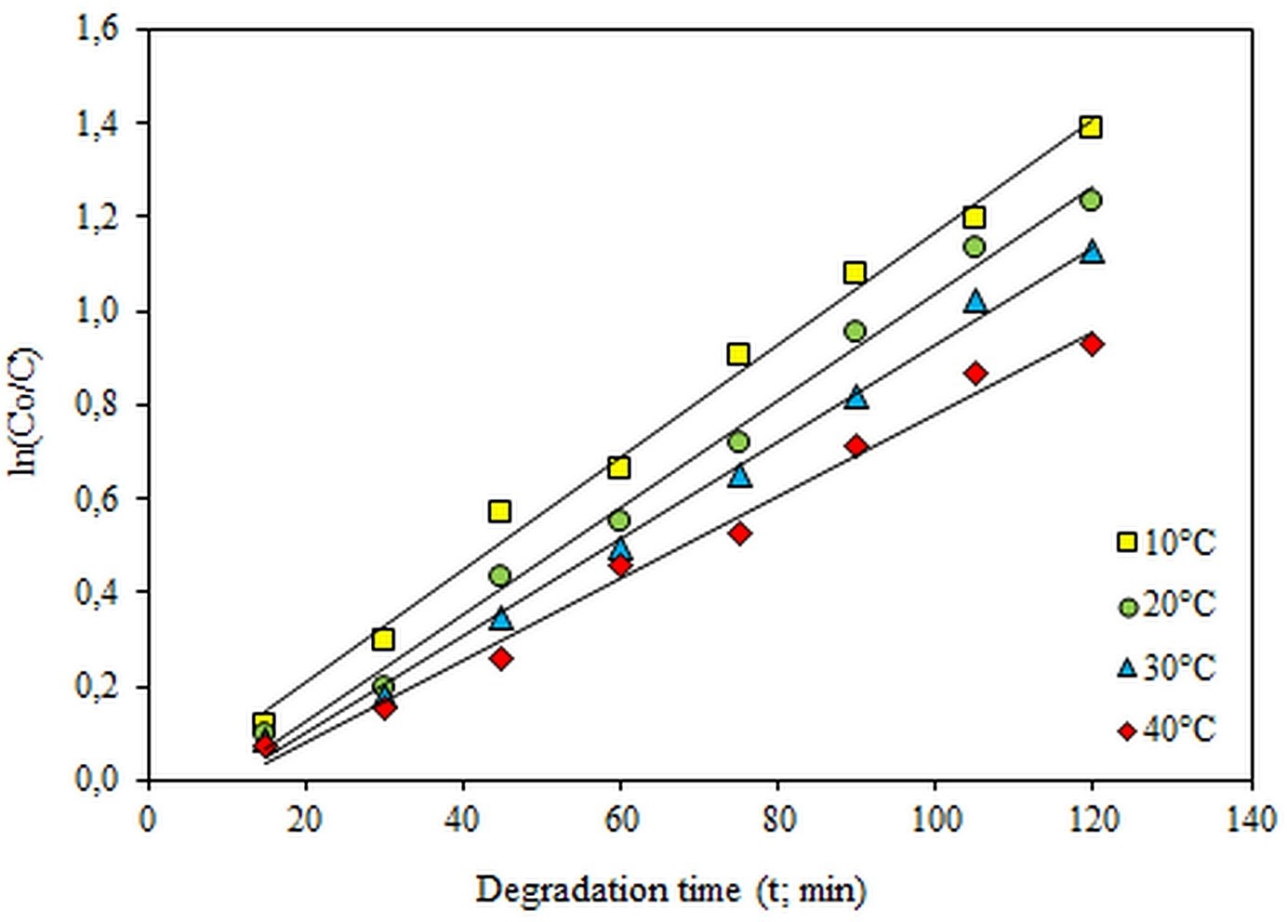
The first‐order kinetic plot of ln (C_o_/C) versus t (2.125 mmol/L Phenol, 8 g/L ZnO, 0.3 L/min air flow rate.

When Figure 6 is examined, it is seen that the data obtained fits the pseudo‐first‐order kinetic model. It is reasonable to state that the findings obtained are consistent when the R^2^ values for phenol, an organic environmental pollutant, are analyzed.

Since the photocatalytic degradation of phenol conforms to the pseudo‐first‐order kinetic model, the activation energy (E_A_) can be calculated using the pseudo‐first‐order kinetic model rate constants. The results obtained by linearizing the Arrhenius equation in Eq. 9 below were plotted, and the activation energy of the photocatalytic degradation of phenol was calculated as 8.24 kJ/mol.

(9) $$k=A\;.\;e^{-\;\frac{E_{A}}{\mathrm{RT}}}$$

## Conclusions

In conclusion, this study evaluated the photocatalytic degradation of phenol and the measurement of TOC using a ZnO photocatalyst, demonstrating the feasibility of employing RSM. The experimental outcomes were effectively modeled using a quadratic equation for the response variables (phenol and TOC) in relation to initial phenol concentration, catalyst concentration, airflow rate, and degradation time. The accuracy of the model was tested for an initial phenol concentration of 100 mg/L under optimum conditions, including a catalysis concentration of 8 g/L, an airflow rate of 0.3 L/min, and a degradation time of 105 min. The model predicted degradation efficiencies of 79.66 % and 61.05 % for phenol and TOC, respectively. Experimental investigations revealed that under optimized conditions, the highest removals achieved for phenol and TOC were 77.15 % and 59.87 %, respectively. The model, developed with predicted results, was experimentally validated under the same optimum conditions. It was observed that the obtained results closely aligned with those predicted by the software, affirming the reliability of the model. Moreover, it was determined that the Langmuir‐Hinshelwood equation‘s regular pattern and the phenol photocatalytic degradation rates were in good agreement.

## Experimental Section

### Chemicals

Zinc oxide (ZnO) (purity≥99.0 %) used in this study has a surface area of 1.48 m^2^/g and was supplied by TEKKIM Chemistry (Turkey). Phenol (purity≥99.0 %) was also purchased from Sigma‐Aldrich. All chemicals used were of analytical grade, and none of the compounds were further purified. Millipore Direct‐Q 3 Ultrapure water was used throughout the study.

### Photo degradation studies

**Figure 7:**
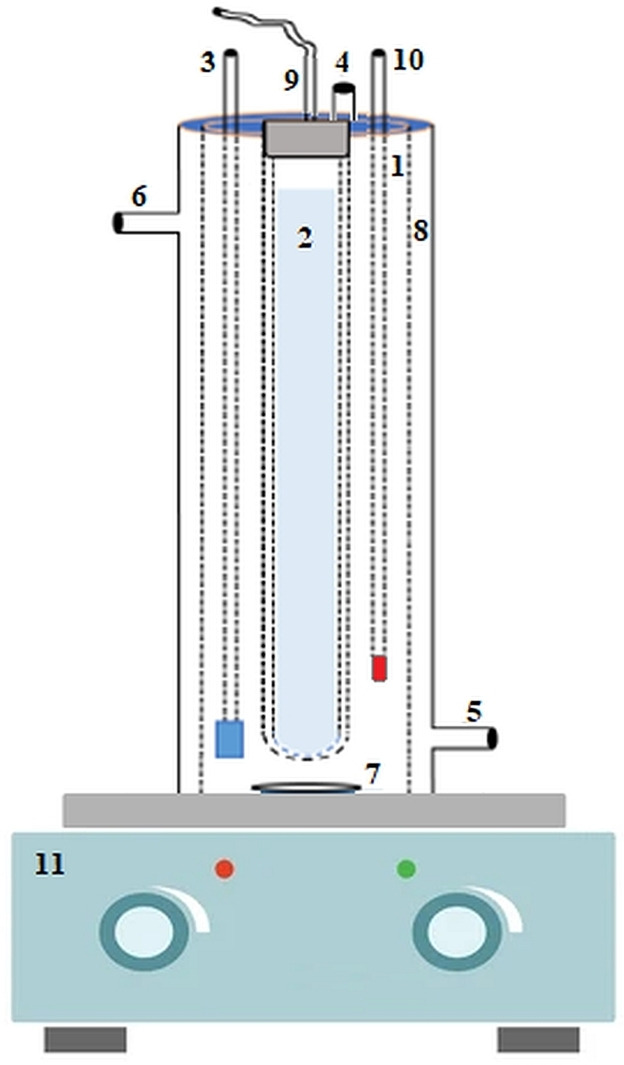
Schematic diagram of the photocatalytic reactor, (1‐ Cylindrical photocatalytic reactor; 2‐ UV−C lamp; 3‐ Air supply; 4‐ Sample port; 5‐ Cooling jacket water inlet; 6‐ Cooling jacket water outlet; 7‐ Magnetic stir bar; 8‐ Cooling Jacket; 9‐ Power source; 10‐ Thermometer; 11‐Magnetic stirrer).

The photocatalytic degradation of phenol was carried out in a custom‐built reactor system with a volume of 500 mL (Figure 7). The experimental apparatus consisted of two nested glass cylinders. The inner glass cylinder is quartz and protects the UV−C light. The outer glass is made of Pyrex glass as a cooling jacket and allows working at a constant temperature. The light source was a 21 Watt UV−C lamp, which was positioned in the center of the photo reactor. An immersed gas distributor from the top of the reactor provided the airflow rate. The temperature change of the reactor was monitored by placing the thermometer at the top. The reactor was placed on a magnetic stirrer to ensure heterogeneous mixing.
(10)

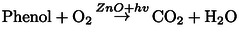




In the basic photocatalytic reaction mechanism, dissolved oxygen is needed to produce sufficient amounts of O_2_* radicals in the reaction medium. Moreover, in heterogeneous photocatalytic degradation processes, very good distribution and mixing of the solid photocatalyst (ZnO) in the aqueous phase is important for degradation efficiency. For this reason, the air fed to the photo reactor is of critical importance and has been evaluated as an effective parameter.^[55]^

After adding 500 mL of phenol solution and ZnO catalyst to the reactor system, the airflow rate on the flow meter was adjusted and supplied into the reactor. The solution was continuously stirred for 15min in dark conditions to attain an adsorption‐desorption state before switching on the light. The solution was stirred continuously at 600 rpm by switching on the light of the UV lamp. Samples were taken from the sampling port at the top of the reactor at certain time intervals, first centrifuged, and then analyzed by taking samples from the solution centrifuged with a syringe. The phenol and TOC removal efficiencies in the samples were analyzed using a UV‐Vis Spectrophotometer (Shimadzu UV‐1800) and TOC−L instrument (Shimadzu), respectively. The following equations (11 and 12) were used for the phenol and TOC removal efficiencies,

(11) $$The\;phenol\;removal\;efficiency;\;\%=\left(\frac{C_{0}-C_{t})}{C_{0}}\right).100$$

(12) $$TOC\;removal\;efficiency;\;\%=\left(\frac{C_{0,TOC}-C_{t,TOC})}{C_{0,TOC}}\right).100$$

where C_o_ is the initial concentration of the phenol or TOC and C_t_ is the final concentration of the phenol or TOC at the time (t).

**Figure 8:**
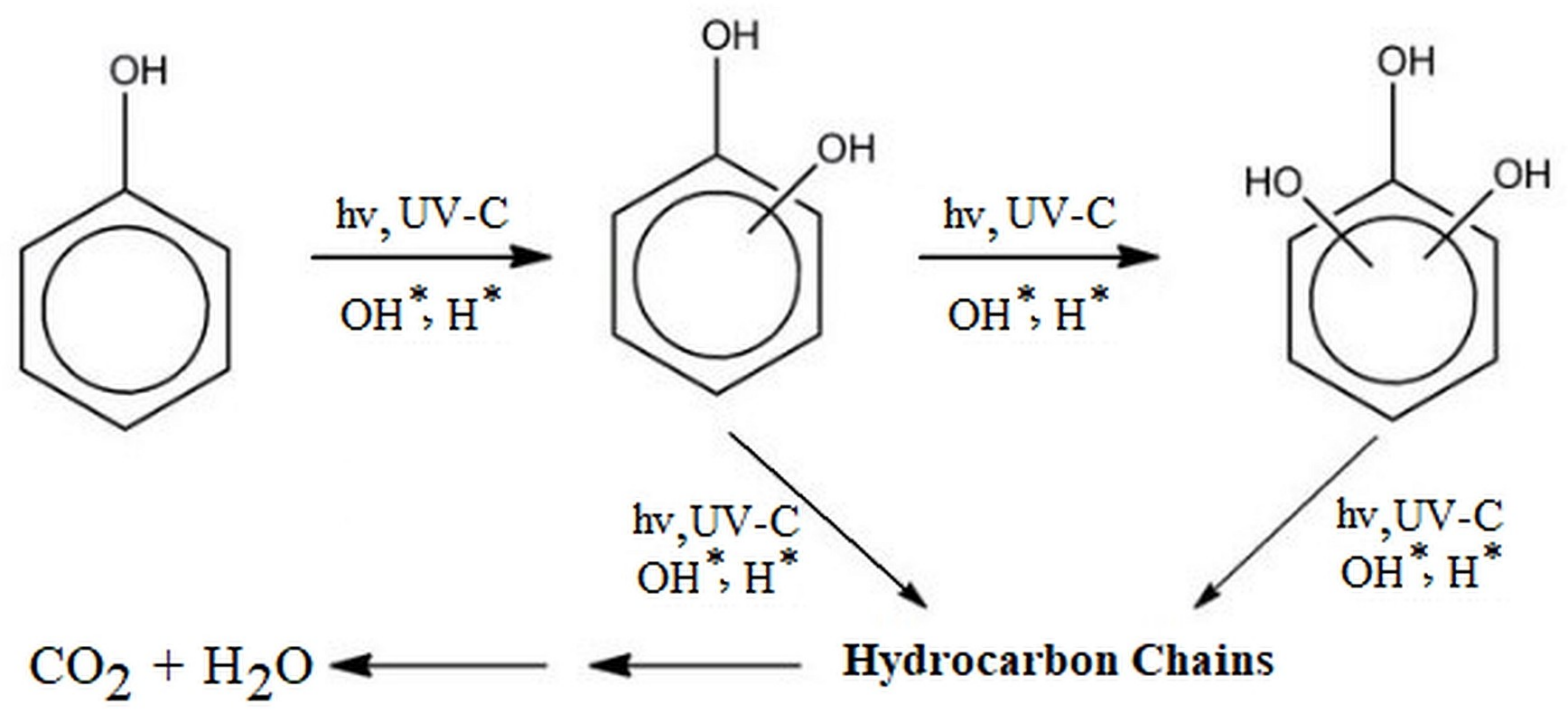
The degradation mechanism of phenol in photocatalysis.

As foreseen in Figure 8,^[56]^ it is a very optimistic approach to assume that the phenol selected as an organic pollutant in the photocatalytic degradation of phenol in the presence of a UV−C lamp is completely broken down into end products (CO_2_ and H_2_O). When considered more realistically, in the reaction environment of phenol degradation, there will be various hydrocarbon compounds with organic content as well as end products (CO_2_ and H_2_O). Initially, spectrophotometric analysis of phenol in the medium will give an idea, but will not reflect reality. In order to accurately reveal the degradation efficiency, it is considered necessary to monitor the degradation of the total organic content in the solution medium by determining TOC.

### Optimization of UV/phenol Process Conditions by RSM

RSM used to analyze the significance of different process variables, is a set of statistical and mathematical approaches. In this study, RSM was used to evaluate the effects of four independent parameters, such as the initial concentration of phenol (A), catalyst concentration (B), airflow rate (C), and degradation time (D). Multivariate regression analysis, modeling, and optimization were performed via CCD based on RSM using Design‐Expert Software 10. Using this technique, each experimental component had a minimum or low level, a center or medium level, and a high or maximum level. (Table 5). The ranges corresponding to the independent parameters used in the CCD were selected from the preliminary experimental results. The experimental design, performed by applying CCD, consists of a total of thirty experiments, six of which are central points, eight are axial and sixteen are factorial. Table 6 presents the 30 experimental runs created using the RSM‐based CCD method. The data of the predicted model were analyzed for significance and suitability using Analysis of Variance (ANOVA).

**Table 5:** Ranges and levels of the independent variables for phenol degradation.

Independent variables (Unit)	Symbol		Level			
		−2.0	−1.0	0.0	+1.0	+2.0
Initial phenol concentration, mg/L	A	50	100	150	200	250
Catalyst concentration, g/L	B	2	4	6	8	10
Airflow rate, L/min	C	0	0.1	0.2	0.3	0.4
Degradation time, min	D	15	45	75	105	135

**Table 6:** Design matrix for factors and responses in experiments at various factor levels.

Run No.	(A) Initial phenol concentration (mg/L)		(B) Catalyst concentration, (g/L)		(C) Airflow rate, (L/min)		(D) Degradation time (min)		Phenol Degradation, %		TOC Removal, %	
							Exp.		Pred.	Exp.		
1	100	−1	4	−1	0.1	−1	45	−1	20.66	22.18	20.20	17.81
2	200	+1	4	−1	0.1	−1	45	−1	8.350	4.750	10.23	7.870
3	100	−1	8	+1	0.1	−1	45	−1	24.21	23.63	23.88	21.92
4	200	+1	8	+1	0.1	−1	45	−1	10.03	10.94	9.250	11.32
5	100	−1	4	−1	0.3	+1	45	−1	28.97	32.88	26.96	28.39
6	200	+1	4	−1	0.3	+1	45	−1	10.21	10.17	12.75	12.27
7	100	−1	8	+1	0.3	+1	45	−1	20.30	28.79	25.23	28.55
8	200	+1	8	+1	0.3	+1	45	−1	10.51	10.82	11.56	11.76
9	100	−1	4	−1	0.1	−1	105	+1	65.05	65.06	43.65	43.80
10	200	+1	4	−1	0.1	−1	105	+1	23.67	16.58	24.38	19.95
11	100	−1	8	+1	0.1	−1	105	+1	69.98	71.43	49.96	49.33
12	200	+1	8	+1	0.1	−1	105	+1	31.28	27.69	25.90	24.82
13	100	−1	4	−1	0.3	+1	105	+1	78.34	78.83	62.66	59.48
14	200	+1	4	−1	0.3	+1	105	+1	24.16	25.07	27.14	29.44
15	100	−1	8	+1	0.3	+1	105	+1	75.74	79.66	58.34	61.05
16	200	+1	8	+1	0.3	+1	105	+1	30.76	30.64	29.08	30.36
17	50	−2	6	0	0.2	0	75	0	97.99	89.24	58.85	58.75
18	250	+2	6	0	0.2	0	75	0	15.78	22.79	17.24	18.11
19	150	0	2	−2	0.2	0	75	0	20.78	23.58	23.42	27.52
20	150	0	10	+2	0.2	0	75	0	35.14	30.60	35.89	32.55
21	150	0	6	0	0.0	−2	75	0	6.790	13.13	4.990	9.930
22	150	0	6	0	0.4	+2	75	0	34.86	26.78	30.22	26.05
23	150	0	6	0	0.2	0	15	−2	4.970	0.370	6.085	5.790
24	150	0	6	0	0.2	0	135	+2	60.20	63.04	49.30	50.37
25	150	0	6	0	0.2	0	75	0	26.13	25.23	27.17	26.81
26	150	0	6	0	0.2	0	75	0	27.35	25.23	25.91	26.81
27	150	0	6	0	0.2	0	75	0	25.03	25.23	29.38	26.81
28	150	0	6	0	0.2	0	75	0	23.13	25.23	27.42	26.81
29	150	0	6	0	0.2	0	75	0	24.26	25.23	24.91	26.81
30	150	0	6	0	0.2	0	75	0	25.45	25.23	26.04	26.81

## Declarations

**Ethics approval** Not applicable.

**Consent to participate** Not applicable.

**Consent for publication** Not applicable.

## Author Contributions

**Meliha SELOGLU**: performed the experiment, and discussed the results; **Ramazan ORHAN**: contributed to the collection of literature and writing the manuscript; **Veyis SELEN**: contributed to the design and the analysis of the results. All the authors read and approved the final manuscript; **Gülbeyi DURSUN**: set the idea and guidelines for conducting the experiment.

## Conflict of interests

The authors declare no competing interests.

1

## Data Availability

The data that support the findings of this study are available from the corresponding author upon reasonable request.
